# Shifting attitudes toward suicide over time: A latent profile analysis using the Korea National Suicide Survey

**DOI:** 10.3389/fpsyt.2023.1124318

**Published:** 2023-03-01

**Authors:** Hyunju Lee, Sang Jin Rhee, Min Ji Kim, Christopher Hyung Keun Park, Jeong Hun Yang, Kyunghoon Son, Jong-Ik Park, Yong Min Ahn

**Affiliations:** ^1^Department of Neuropsychiatry, Seoul National University Hospital, Seoul, Republic of Korea; ^2^Department of Psychiatry, Seoul National University College of Medicine, Seoul, Republic of Korea; ^3^Department of Psychiatry, Asan Medical Center, Seoul, Republic of Korea; ^4^Department of Psychiatry, Kangwon National University School of Medicine, Chuncheon, Republic of Korea; ^5^Institute of Human Behavioral Medicine, Seoul National University Medical Research Center, Seoul, Republic of Korea

**Keywords:** suicide in Korea, attitudes to suicide, Korea National Suicide Survey, latent profile analysis, moderation model, Werther effect

## Abstract

**Introduction:**

South Korea has a high suicide rate, and changes in sociodemographic factors can further increase the rate. This study aims to (1) classify participants using the Attitudes toward Suicide Scale (ATTS) through latent profile analysis (LPA), (2) identify and compare the associations between sociodemographic factors with the ATTS in two survey years (2013, 2018), and (3) determine the moderating effect of survey year.

**Methods:**

Six sub-factors of the ATTS were used for LPA with a total of 2,973 participants. Sociodemographic characteristics were compared between groups, and multinomial logistic regression was conducted for each survey year. A moderation analysis was conducted with the survey year as moderator.

**Results:**

LPA identified three groups of attitudes toward suicide: incomprehensible (10.3%), mixed (52.8%), and permissive (36.9%). The proportion of permissive attitudes increased from 2013 (32.3%) to 2018 (41.7%). Participants reporting suicidal behavior were more likely to be in the mixed and permissive groups than the incomprehensible group in both years. People reporting no religious beliefs were associated with the permissive group in the two survey years. The influence of education and income levels on groups differed by survey year.

**Discussion:**

There were significant changes between 2013 and 2018 in attitudes toward suicide in the Korean population.

## 1. Introduction

According to the age-standardized suicide rate among Organization for Economic Cooperation and Development (OCED) countries, South Korea's rate is 23.5 per 100,000 people, which is more than twice the average rate of 10.9 per 100,000 across the 38 OECD countries (1). In 2020, suicide was the leading cause of death for people aged 10–39, and the suicide rate among people in their 60s and older was the highest among OECD countries (1, 2). Suicide, thus, remains a major health problem in South Korea. After reaching its peak in 2011, the suicide rate slowly decreased until 2017 due to various efforts by the government and society, for example, the activation of a government project named “Prevention of Suicide and Proliferation of a Culture of Respect for Life,” which is a basic plan of countermeasures to prevent suicide (2, 3). However, the rate rebounded in 2018 and 2019 due to cases of celebrity suicide and worsening economic conditions for low-income people (2, 3). The number of deaths by suicide decreased in 2020 in the context of the COVID-19 pandemic and the reduced visibility of celebrity and copycat suicides compared to the previous year (2, 4). The suicide rate is, therefore, affected by complex factors, with the socio-cultural background playing a significant role (5).

One study of the U.S. population indicated that the culture has shifted toward tolerance of suicide (6). Changes in population composition over time accounted for ~50% of the increase in suicide acceptability between the 1980s and 2010s in the U.S. (6). Over time, Americans became more educated, were more likely to be single and to live in urban areas, and became less religious. These shifts were related to higher levels of tolerance toward suicide. Similar changes can be observed in the Korean population. The lifetime single-marital status rate increased from 8.0% in 2015 to 16.6% in 2020 (7). Further, the rate of the adult population who graduated college more than doubled from 23.8% in 2000 to 50.7% in 2020 (8). Higher-educated, single individuals have been linked to permissive attitudes toward suicide (6, 9). Meanwhile, the number of religious people, who are known to have an attitude that suicide is incomprehensible, was 54% in 2004 but decreased to 50% in 2014 and 40% in 2021 (10, 11). Additionally, news articles reporting celebrity suicides are accessible online, increasing the risk of copycat suicides among high-risk groups (12–14), and unregulated reports could make individuals perceive suicide as their right (12–15). The risk of suicide-acceptive attitudes has been increasing, while the protective factors are gradually decreasing.

Individual attitudes toward suicide are related to suicide intensity and behavior (16–18). To summarize, understanding the risks and protective factors related to the perception of suicide is important in establishing suicide prevention policies to prepare for future demographic changes. Previous studies on attitudes toward suicide have mainly compared specific population groups and factors, such as country, age, sex, and bereavement (16, 18, 19). We previously compared attitudes toward suicide between people who had experienced the suicide of an acquaintance and those who had not (17). However, few studies have classified attitudes toward suicide among different groups and investigated the correlation of sociodemographic factors. Therefore, this study aimed to identify attitudes toward suicide in the Korean population using the Attitudes toward Suicide Scale (ATTS). In addition, this study attempted to determine the sociodemographic factors in groups with different attitudes in both survey years (2013 and 2018). Furthermore, the moderating effect of survey year was analyzed to explore whether the influence of each sociodemographic factor changed over time.

## 2. Materials and methods

### 2.1. Participants

The participants' details and data collection methods have been described in our previous articles (17, 20). South Korean adults aged 19–75 voluntarily participated after being informed about the study (20). Data were conducted through face-to face interviews, and a total of 2,973 people were included in the final analysis (1,473 in 2013 and 1,500 in 2018) (20). There were no missing values in the final analysis. Written consent was waived as the survey involved minimal risk. The survey was monitored and approved by the Institutional Review Board of Seoul National University Hospital (IRB No. 1405-019-577) and Kangwon National University Hospital (IRB No. KNUH-2013-06-007-001). The study was conducted in accordance with the Declaration of Helsinki.

### 2.2. Assessments

#### 2.2.1. The attitudes toward suicide scale

The ATTS was developed by Renberg and Jacobsson (21) and comprises 37 items rated on a five-point Likert scale. It was used to evaluate the participants' attitudes toward suicide in 2013 and 2018. The ATTS has been frequently used in large-scale studies due to its relatively small number of questions and simplicity compared to other scales (16, 18, 21, 22). Although it has a ten-factor structure (21), six factors with a reliability of 0.4 or more were selected (suicide as right: 0.67, incomprehensibility: 0.59, resignation: 0.64, relation-caused: 0.47, preventability: 0.43, and normal-common: 0.41) (23). The remaining four factors (non-communication, tabooing, suicidal process, and preparedness to prevent) showed very low reliability (Cronbach's alpha < 0.4) with the present data (23). Therefore, these factors were excluded, and the following six factors were used for a latent profile analysis (LPA): suicide as a right, incomprehensibility, resignation, relation caused, normal common, and preventability.

#### 2.2.2. Sociodemographic characteristics

Sociodemographic characteristics were recorded, including age, sex, education, marital status, monthly income, region, and religion. Additional questions related to suicide examined suicidal behavior (“Have you ever had any suicide-related behavior?”—no idea/idea only/plan or attempt), experience of suicidal loss among acquaintances (“Is there anyone around you who has committed suicide?”—yes/no), and interest in media reporting suicide (“Are you interested in articles reporting suicide?”—yes/no).

### 2.3. Statistical analyses

To classify groups according to Korean's attitudes toward suicide, a LPA was conducted on data from 2,793 participants using Mplus 8.0 (24). The optimal number of latent classes was determined using the Bayesian Information Criterion (BIC), sample-size adjusted BIC (saBIC), entropy, Lo-Mendell-Rubin Likelihood Ration (LMR-LR), and Bootstrapped Likelihood Ratio Test (BLRT). Analysis started with one class, and as additional classes were added, the fit indices of each model were evaluated until the optimal number of latent classes was confirmed.

Categorical variables were analyzed using Pearson's chi-squared test utilizing the *post-hoc* analysis with “chisq.posthoc.test” R package (25, 26). The following analysis was performed with IBM SPSS Statistics version 25.0 (SPSS Inc., Chicago, IL, USA). ANOVA with Turkey's multiple comparison was conducted for continuous variables to compare the classified groups. In order to identify the effect of sociodemographic factors on the groups, multinomial logistic regression analysis was performed for each survey year. All sociodemographic factors were entered into the logistic regression analysis. To determine the moderating effect of survey year on each demographic factor, Hayes' PROCESS version 3.5 macro for SPSS (Model 1) was performed adjusting age and sex as covariates (27), and additionally adjusting all other sociodemographic factors. The statistically significant level was a two-tailed *p*-value < 0.05.

## 3. Results

### 3.1. Comparison of sociodemographic characteristics between survey years

When comparing the sociodemographics between survey years, the frequency of education, monthly income, religion, interest in media reporting suicide, and suicide behavior showed significant differences (Table 1). First, when comparing the frequency of education between 2013 and 2018, < high school was lower in 2018 (*n* = 190, 12.7%) than in 2013 (*n* = 318, 21.6%), and the frequency of college or graduate school was higher in 2018 (*n* = 735, 49.0%) than in 2013 (*n* = 549, 37.3%). The frequency of low-income was lower in 2018 (*n* = 217, 14.5%) than in 2013 (*n* = 408, 27.7%), and the frequency of high-income was higher in 2018 (*n* = 649, 43.3%) than in 2013 (*n* = 372, 25.3%). The frequency of believing in a religion was lower in 2018 (*n* = 633, 42.2%) than in 2013 (*n* = 743, 50.4%), and the frequency of having interest in the media reporting suicide was lower in 2018 (*n* = 622, 41.5%) than in 2013 (*n* = 681, 46.2%). There was a statistically significant difference between survey years when comparing the frequency of suicide behavior (*p* = 0.040), although this was not significant after multiple comparisons.

**Table 1:** Comparison of sociodemographic characteristics by survey year.

	**2013 (*n* = 1,473)**	**2018 (*n* = 1,500)**	**Statistics**	***p*-value**
**Age**, ***n*** **(%)**			7.0	0.136
19–29	244 (16.6)	261 (17.4)		
30–39	313 (21.2)	266 (17.7)		
40–49	319 (21.7)	325 (21.7)		
50–59	286 (19.4)	326 (21.7)		
≥60	311 (21.1)	322 (21.5)		
**Sex**, ***n*** **(%)**			2.3	0.131
Male	680 (46.2)	734 (48.9)		
Female	793 (53.8)	766 (51.1)		
**Education year**, ***n*** **(%)**			59.8	**< 0.001**
< High school	**318 (21.6)**	**190 (12.7)**		
High school	606 (41.1)	575 (38.3)		
College or graduate school	**549 (37.3)**	**735 (49.0)**		
**Monthly income, 10**^4^ **won**, ***n*** **(%)**			135.9	**< 0.001**
< 200	**408 (27.7)**	**217 (14.5)**		
200–400	693 (47.0)	634 (42.3)		
>400	**372 (25.3)**	**649 (43.3)**		
**Marital status**, ***n*** **(%)**			4.8	0.089
Single	302 (20.5)	356 (23.7)		
Married	1,052 (71.4)	1,036 (69.1)		
Divorce or bereavement	119 (8.1)	108 (7.2)		
**Religion**, ***n*** **(%)**			20.3	**< 0.001**
Yes	743 (50.4)	633 (42.2)		
No	730 (49.6)	867 (57.8)		
**Exposure to suicidal loss**, ***n*** **(%)**			0.1	0.703
Yes	146 (9.9)	155 (10.3)		
No	1,327 (90.1)	1,345 (89.7)		
**Interest in media reporting suicide**, ***n*** **(%)**			6.9	**0.009**
Yes	681 (46.2)	622 (41.5)		
No	792 (53.8)	878 (58.5)		
**Suicide behavior**, ***n*** **(%)**			6.4	**0.040**
No idea	1,146 (77.8)	1,215 (81.0)		
Idea only	69 (4.7)	48 (3.2)		
Plan or attempt	258 (17.5)	237 (15.8)		

Statistically significant differences were analyzed by Pearson's chi-square test. Multiple comparison was performed by “chisq.posthoc.test” R packages with Bonferroni correction. **Boldface** values are significant after multiple comparison with Bonferroni correction.

### 3.2. Subtypes of attitudes toward suicide

Table 2 shows the fit indices for the LPA models. The number of appropriate classes was determined based on fit indices, proportion rate, and information the classified groups presented (28). As the number of classified groups increased, the values of BIC and saBIC decreased, with the Class 5 model showing the lowest value. However, this model was excluded because it included a group with less than a 5% proportion (28). A Class 3 model showed a significant LMR-LR *p*-value (*p* < 0.001), which was not significant in the Class 4 model (*p* = 0.083). In addition, the entropy of the Class 3 model was 0.713, the highest among the five models. As a result, the Class 3 model was selected as the most appropriate model. The average latent class posterior probabilities (Table 3), which reflect the accuracy of predicting individual's class membership, were between 0.80 and 0.90 (within the acceptable range) (28, 29).

**Table 2:** Comparison between fit indices of latent profile analysis (*n* = 2,973).

**Model**	**BIC**	**saBIC**	**Entropy**	**LMR-LR *p*-value**	**BLRT *p*-value**	**Proportion (%)**				
						**1**	**2**	**3**	**4**	**5**
1 class	39,054.11	39,015.98	N/A	N/A	N/A	100.0				
2 class	37,058.87	36,998.50	0.702	< 0.001	< 0.001	33.3	66.7			
**3 class**	**36,504.62**	**36,422.01**	**0.713**	**< 0.001**	**< 0.001**	**10.3**	**52.8**	**36.9**		
4 class	36,391.54	36,286.69	0.689	0.083	< 0.001	43.2	40.5	7.5	8.8	
5 class	36,315.26	36,188.17	0.709	0.372	< 0.001	7.2	40.5	3.2	42.8	6.3

BIC, Baysian Information Criterion; saBIC, sample-size adjusted Baysian Information Criterion; LMR-LR, Lo-Mendell-Rubin Likelihood Ratio; BLRT, Bootstrapped Likelihood Ratio Test; N/A, not applicable. The most appropriate model is in boldface.

**Table 3:** Average latent class probabilities for most likely latent class membership (row) by latent class (column).

	**Latent class**		
	**1**	**2**	**3**
**Most likely latent class membership**			
1	0.87	0.13	0.00
2	0.04	0.86	0.10
3	0.00	0.13	0.87

**Figure 1:**
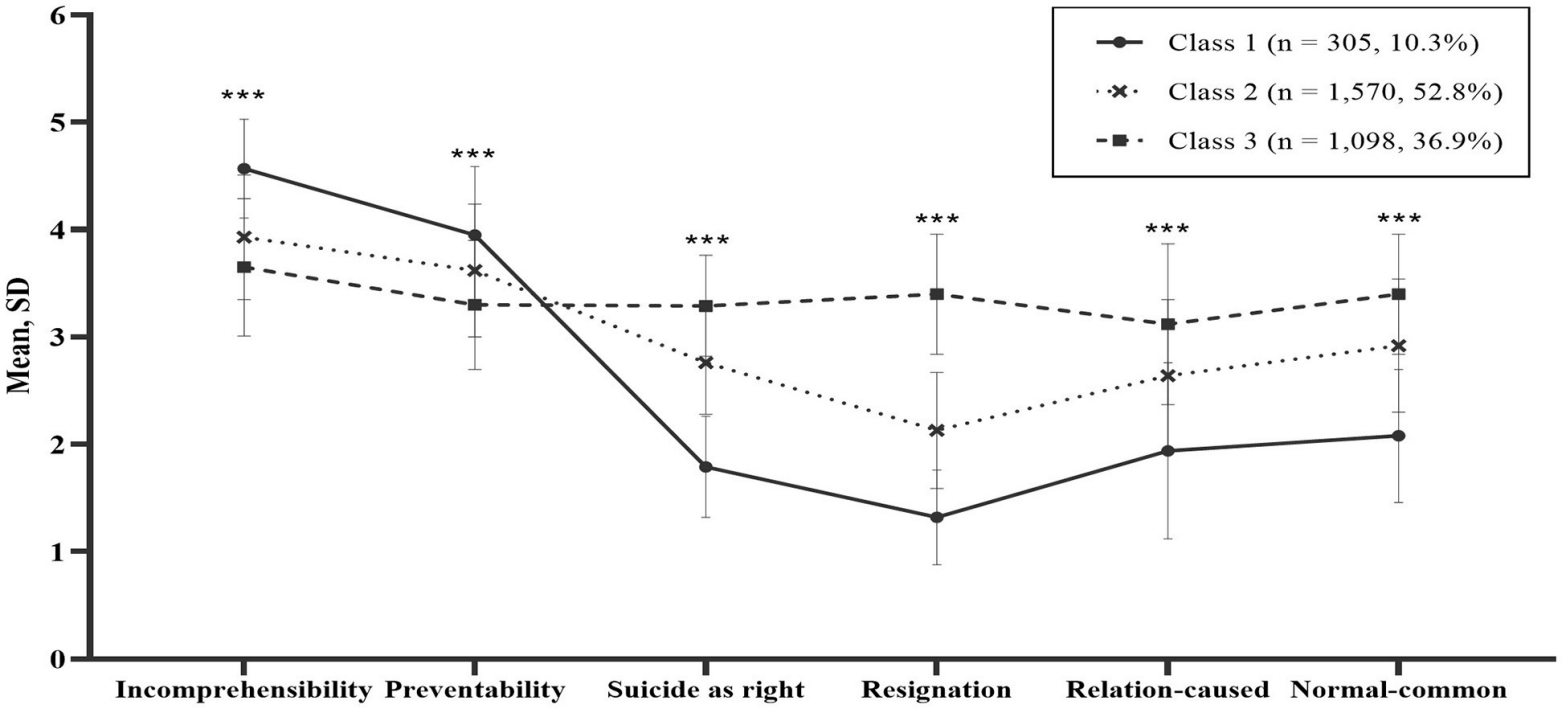
Mean and SD of factors of attitudes toward suicide (ATTS) of 3 class model. ^***^p < 0.001.

The three groups classified by LPA were named according to the characteristics of the sub-scales for six attitudes toward suicide (Figure 1). Compared to the other two groups, Class 1 (10.3%, *n* = 305) had the highest score for incomprehensibility and preventability factors, while factors receptive to suicide (suicide as a right, resignation, relation-caused, and normal common) had the lowest score. Therefore, it was labeled the “incomprehensible” group. Conversely, Class 3 (36.9%, *n* = 1,098) was characterized by the highest score for suicide as a right, resignation, relation-caused, and normal common, while the lowest score was for incomprehensibility and preventability. Therefore, Class 3 was labeled the “permissive” group. Class 2 (52.8%, *n* = 1,570) showed the middle scores of Classes 1 and 3 in all six factors and was named the “mixed” group (Figure 1).

### 3.2. Comparisons of sociodemographic characteristics between classes

**Figure 2:**
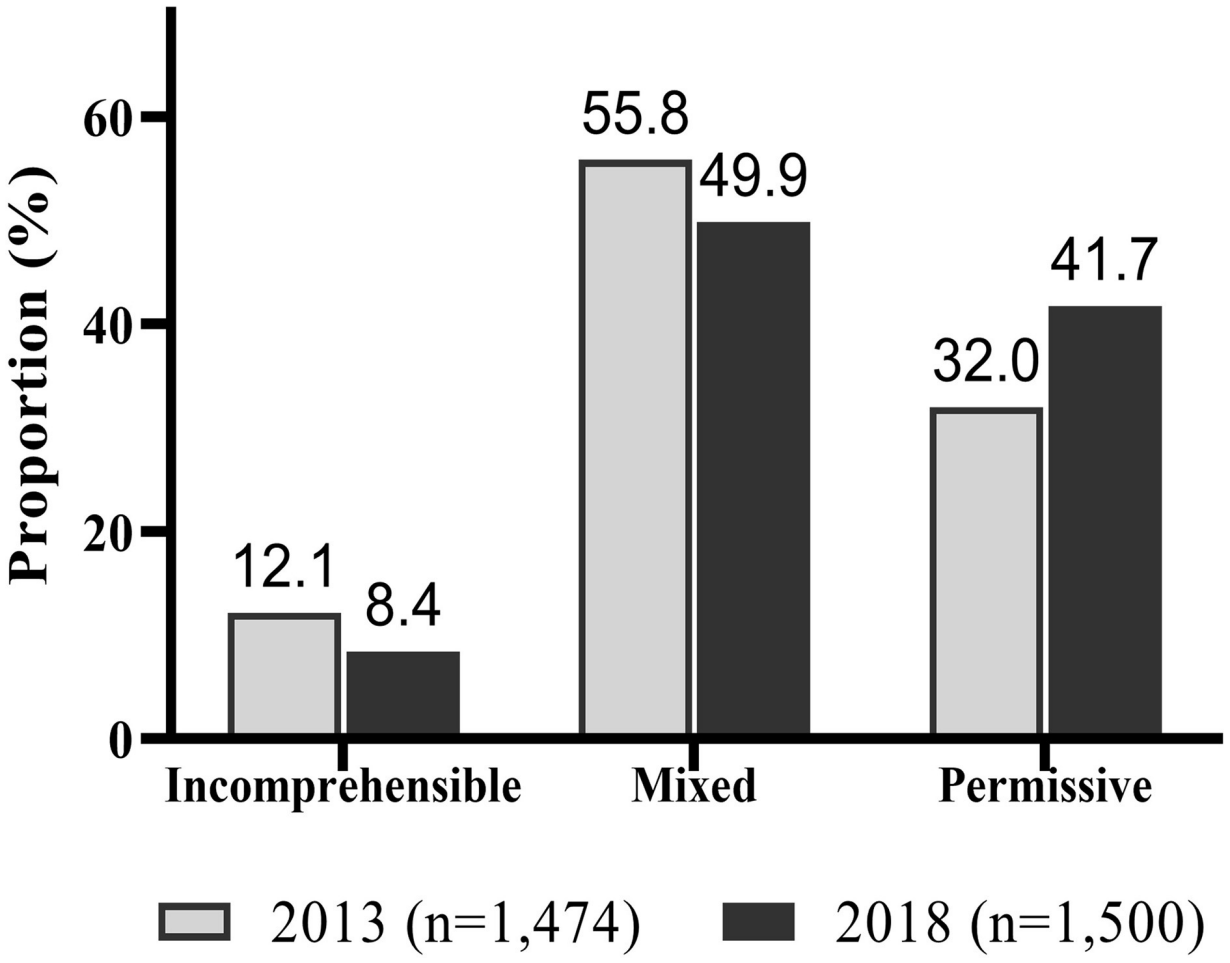
Comparison of frequency of classified groups by survey year (2013, 2018).

As a result of analyzing the frequency of groups classified by survey year, the proportions of the groups classified as incomprehensible [12.1% (2013), 8.4% (2018)] and mixed [55.8% (2013), 49.9% (2018)] were higher in 2013 than 2018, and the proportion of groups classified as permissible was higher in 2018 (41.7%) than in 2013 (32.0%) (Pearson's χ^2^ = 34.06, *p* < 0.001) (Figure 2).

The results of comparing sociodemographic characteristics between the three groups in each survey year are presented in Table 4. Monthly income, religion, and suicide factors showed significant differences between groups in 2013 and 2018. In 2013, the proportion of low monthly income (< 200, 10^4^ won) was higher in the permissive group (32.6%), and medium monthly income (200–400, 10^4^ won) was higher in the incomprehensible group (57.5%) than in other groups. The results in 2018 were not significant after multiple comparison with Bonferroni correction. Participants with religious beliefs were less often classified as permissive (44.5%) in 2013 and more often classified into the incomprehensible group (55.6%) in 2018. Among participants with no suicidal ideas, the proportions of those in the incomprehensible group [89.4% (2013), 91.3% (2018)] were higher and the proportions of those in the permissive group [70.6% (2013), 76.8% (2018)] were lower than in the other groups. In contrast, among participants with suicidal plans or attempts, the proportions of those in the incomprehensible group [8.4% (2013), 7.1% (2018)] were lower in the permissive [22.2% (2013)] group. There were no differences between the three groups in terms of the age, sex, marital status, exposure to suicidal loss, or interest in media reporting suicide factors (Table 4).

**Table 4:** Comparison of sociodemographic characteristics between the three classes by survey year.

	**2013**					**2018**				
	**Incomprehensible group (*****n*** = **179)**	**Mixed group (*****n*** = **822)**	**Permissive group (*****n*** = **472)**	**Statistics**	***p*** **value**	**Incomprehensible group (*****n*** = **126)**	**Mixed group (*****n*** = **748)**	**Permissive group (*****n*** = **626)**	**Statistics**	***p*** **-value**
**Age**, ***n*** **(%)**				14.2	0.076				8.3	0.404
19–29	20 (11.2)	142 (17.3)	82 (17.4)			14 (11.1)	126 (16.8)	121 (19.3)		
30–39	48 (26.8)	175 (21.3)	90 (19.1)			27 (21.4)	127 (17.0)	112 (17.9)		
40–49	41 (22.9)	190 (23.1)	88 (18.6)			32 (25.4)	161 (21.5)	132 (21.1)		
50–59	36 (20.1)	146 (17.8)	104 (22.0)			30 (23.8)	169 (22.6)	127 (20.3)		
≥60	34 (19)	169 (20.6)	108 (22.9)			23 (18.3)	165 (22.1)	134 (21.4)		
**Sex**, ***n*** **(%)**				3.5	0.178				2.1	0.353
Male	89 (49.7)	362 (44.0)	229 (48.5)			56 (44.4)	360 (48.1)	318 (50.8)		
Female	90 (50.3)	460 (56.0)	243 (51.5)			70 (55.6)	388 (51.9)	308 (49.2)		
**Education**, ***n*** **(%)**				13.7	**0.008**				6.2	0.184
< High school	27 (15.1)	168 (20.4)	**123 (26.1)**			15 (11.9)	109 (14.6)	66 (10.5)		
High school	71 (39.7)	343 (41.7)	192 (40.7)			45 (35.7)	289 (38.6)	241 (38.5)		
College or graduate school	81 (45.3)	311 (37.8)	157 (33.3)			66 (52.4)	350 (46.8)	319 (51.0)		
**Monthly income, 10**^4^ **won**, ***n*** **(%)**				16.2	**0.003**				10.4	**0.035**
< 200	37 (20.7)	217 (26.4)	**154 (32.6)**			24 (19.0)	118 (15.8)	75 (12.0)		
200–400	**103 (57.5)**	380 (46.2)	210 (44.5)			58 (46.0)	296 (39.6)	280 (44.7)		
>400	39 (21.8)	225 (27.4)	108 (22.9)			44 (34.9)	334 (44.7)	271 (43.3)		
**Employment**										
Employed										
Unemployed										
**Marital status**, ***n*** **(%)**				6.2	0.182				6.3	0.178
Single	28 (15.6)	165 (20.1)	109 (23.1)			22 (17.5)	169 (22.6)	165 (26.4)		
Married	137 (76.5)	595 (72.4)	320 (67.8)			92 (73)	526 (70.3)	418 (66.8)		
Divorce or bereavement	14 (7.8)	62 (7.5)	43 (9.1)			12 (9.5)	53 (7.1)	43 (6.9)		
**Religion**, ***n*** **(%)**				9.9	**0.007**				12.3	**0.002**
Yes	97 (54.2)	436 (53.0)	**210 (44.5)**			**70 (55.6)**	320 (42.8)	243 (38.8)		
No	82 (45.8)	386 (47.0)	**262 (55.5)**			**56 (44.4)**	428 (57.2)	383 (61.2)		
**Exposure to suicidal loss**, ***n*** **(%)**				5.8	0.055				1.1	0.590
Yes	10 (5.6)	80 (9.7)	56 (11.9)			11 (8.7)	83 (11.1)	61 (9.7)		
No	169 (94.4)	742 (90.3)	416 (88.1)			115 (91.3)	665 (88.9)	565 (90.3)		
**Interest in media reporting suicide**, ***n*** **(%)**				4.5	0.105				5.4	0.069
Yes	71 (39.7)	396 (48.2)	214 (45.3)			40 (31.7)	317 (42.4)	265 (42.3)		
No	108 (60.3)	426 (51.8)	258 (54.7)			86 (68.3)	431 (57.6)	361 (57.7)		
**Suicide behavior**, ***n*** **(%)**				31.5	**< 0.001**				18.0	**0.001**
No idea	**160 (89.4)**	653 (79.4)	**333 (70.6)**			**115 (91.3)**	619 (82.8)	**481 (76.8)**		
Idea only	4 (2.2)	31 (3.8)	**34 (7.2)**			2 (1.6)	19 (2.5)	27 (4.3)		
Plan or attempt	**15 (8.4)**	138 (16.8)	**105 (22.2)**			**9 (7.1)**	110 (14.7)	118 (18.8)		

Statistically significant differences were analyzed by Pearson's chi-square test. Multiple comparison was performed by “chisq.posthoc.test” R packages with Bonferroni correction. **Boldface** values are significant after multiple comparison with Bonferroni correction.

### 3.3. Results of multinomial logistic regression assessing the three classes in each survey year

Table 5 shows the results of multinomial logistic regression to identify differences in the sociodemographic characteristics of groups in each survey year. To explore the sociodemographic factors affecting acceptive attitudes toward suicide, the incomprehensible group was designated as the reference group. Age, sex, and marital status did not significantly differ among the three classes in 2013 and 2018. Medium monthly income was less likely to be associated with the mixed group in 2013 [OR = 0.60, 95% CI (0.40, 0.91)] and 2018 [OR = 0.60, 95% CI (0.39, 0.93)]. Low monthly income was less likely to be associated with the mixed [OR = 0.42, 95% CI (0.21, 0.83)] and permissive groups [OR = 0.34, 95% CI (0.17, 0.68)] in 2018. Participants who had no religious beliefs were more likely to be in the permissive group in 2013 [OR = 1.63, 95% CI (1.13, 2.35)] and 2018 [OR = 2.00, 95% CI (1.33, 3.00)]. Participants who experienced the suicidal loss of acquaintances were more likely to be in the permissive group in 2013 [OR = 2.22, 95% CI (1.09, 4.53)]. In addition, participants who had been interested in media reports related to suicide were more likely to be in the mixed group [OR = 1.58, 95% CI (1.05, 2.38)] and in the permissive group [OR = 1.52, 95% CI (1.00, 2.32)] in 2018. Participants with suicidal plans or attempts were more likely to be in the mixed group in 2013 [OR = 2.16, 95% CI (1.22, 3.81)] and 2018 [OR = 2.34, 95% CI (1.13, 4.84)] and in the permissive group in 2013 [OR = 3.41, 95% CI (1.90, 6.10)] and 2018 [OR = 3.44, 95% CI (1.66, 7.12)].

**Table 5:** Multinomial logistic regression assessing the three classes in each survey year.

	**2013**				**2018**			
**Incomprehensible group (reference group)**	**Mixed group**		**Permissive group**		**Mixed group**		**Permissive group**	
	**OR (95% CI)**	***p*** **-value**	**OR (95% CI)**	***p*** **-value**	**OR (95% CI)**	***p*** **-value**	**OR (95% CI)**	***p*** **-value**
**Age, year**								
19–29	1 (reference)		1 (reference)		1 (reference)		1 (reference)	
30–39	0.55 (0.28–1.08)	0.083	0.63 (0.31–1.30)	0.212	0.58 (0.25–1.35)	0.206	0.58 (0.25–1.36)	0.211
40–49	0.68 (0.33–1.41)	0.297	0.77 (0.35–1.70)	0.519	0.61 (0.25–1.52)	0.290	0.62 (0.25–1.55)	0.303
50–59	0.48 (0.22–1.06)	0.070	0.87 (0.38–2.02)	0.753	0.65 (0.24–1.73)	0.385	0.65 (0.24–1.76)	0.393
≥60	0.51 (0.22–1.19)	0.117	0.75 (0.30–1.86)	0.531	0.98 (0.32–3.02)	0.975	1.38 (0.44–4.30)	0.578
**Sex**								
Male	0.80 (0.57–1.12)	0.195	0.88 (0.61–1.27)	0.487	1.10 (0.73–1.65)	0.663	1.14 (0.75–1.73)	0.539
Female	1 (reference)		1 (reference)		1 (reference)		1 (reference)	
**Education**								
< High school	**2.20 (1.14–4.25)**	**0.019**	**2.40 (1.20–4.81)**	**0.013**	1.97 (0.82–4.72)	0.129	1.15 (0.47–2.83)	0.757
High school	1.31 (0.88–1.93)	0.183	1.19 (0.78–1.82)	0.423	1.46 (0.88–2.44)	0.143	1.30 (0.77–2.18)	0.328
College or graduate school	1 (reference)		1 (reference)		1 (reference)		1 (reference)	
**Monthly income, 10**^4^ **won**								
< 200	0.85 (0.48–1.50)	0.572	1.15 (0.63–2.10)	0.651	**0.42 (0.21–0.83)**	**0.012**	**0.34 (0.17–0.68)**	**0.002**
200–400	**0.60 (0.40–0.91)**	**0.017**	0.72 (0.46–1.14)	0.159	**0.60 (0.39–0.93)**	**0.021**	0.70 (0.45**–**1.10)	0.121
>400	1 (reference)		1 (reference)		1 (reference)		1 (reference)	
**Marital status**								
Single	1.52 (0.62–3.74)	0.365	2.24 (0.86–5.82)	0.097	1.69 (0.61–4.70)	0.314	1.78 (0.63–5.05)	0.280
Married	1.45 (0.73–2.85)	0.288	1.45 (0.71–2.98)	0.306	1.43 (0.67–3.06)	0.356	1.28 (0.59–2.79)	0.539
Divorce or bereavement	1 (reference)		1 (reference)		1 (reference)		1 (reference)	
**Religion**								
Yes	1 (reference)		1 (reference)		1 (reference)		1 (reference)	
No	1.08 (0.77–1.51)	0.670	**1.63 (1.13–2.35)**	**0.009**	**1.71 (1.15–2.55)**	**0.009**	**2.00 (1.33–3.00)**	**0.001**
**Exposure to suicidal loss**								
Yes	1.79 (0.90–3.56)	0.099	**2.22 (1.09–4.53)**	**0.028**	1.17 (0.60–2.31)	0.646	0.95 (0.47–1.90)	0.879
No	1 (reference)		1 (reference)		1 (reference)		1 (reference)	
**Interest in media reporting suicide**								
Yes	1.35 (0.96–1.89)	0.085	1.12 (0.78–1.61)	0.540	**1.58 (1.05**–**2.38)**	**0.029**	**1.52 (1.00**–**2.32)**	**0.048**
No	1 (reference)		1 (reference)		1 (reference)		1 (reference)	
**Suicide behavior**								
No idea	1 (reference)		1 (reference)		1 (reference)		1 (reference)	
Idea only	1.47 (0.50–4.32)	0.481	**3.24 (1.10**–**9.55)**	**0.033**	1.97 (0.44–8.72)	0.374	3.76 (0.86–16.45)	0.078
Plan or attempt	**2.16 (1.22–3.81)**	**0.008**	**3.41 (1.90–6.10)**	**< 0.001**	**2.34 (1.13–4.84)**	**0.022**	**3.44 (1.66–7.12)**	**0.001**

OR, Odds Ratio; CI, Confidence Interval. **Boldface** values are significant at *p* < 0.05.

### 3.4. The moderation effect of survey year

**Figure 3:**
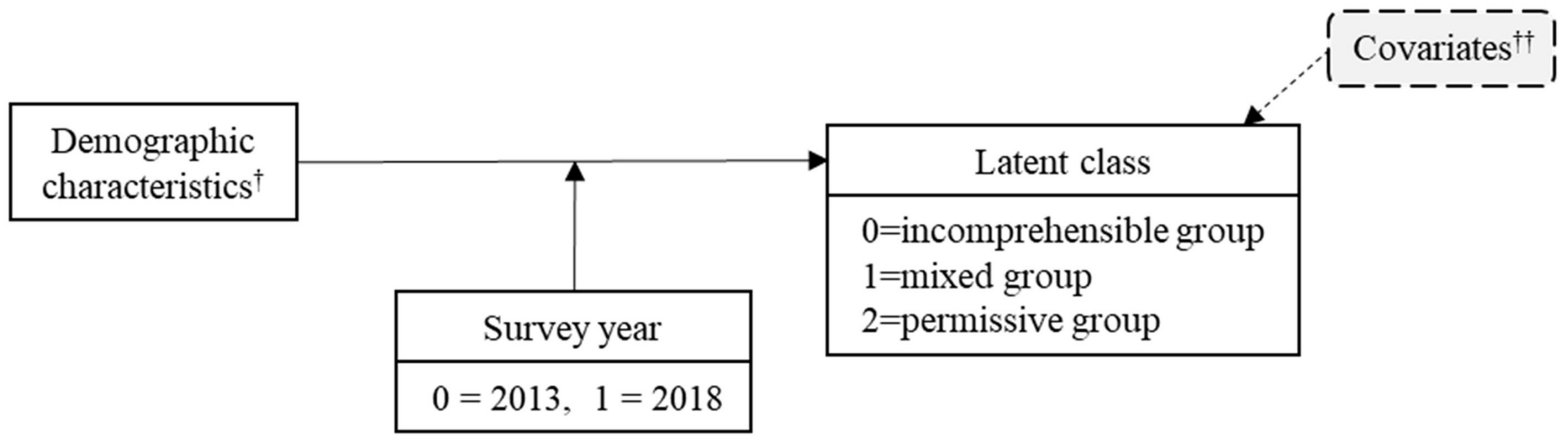
Moderation model. Survey year (2013, 2018) as a moderator in the relationship between each sociodemographic characteristics and latent class. ^†^ (a) Education, (b) Monthly income, (c) Marital status, (d) Religion, (e) Experience of suicidal loss, (f) Interest in media reporting suicide, (g) Suicidal behavior. ^†^^†^ Covariates: age, sex.

**Figure 4:**
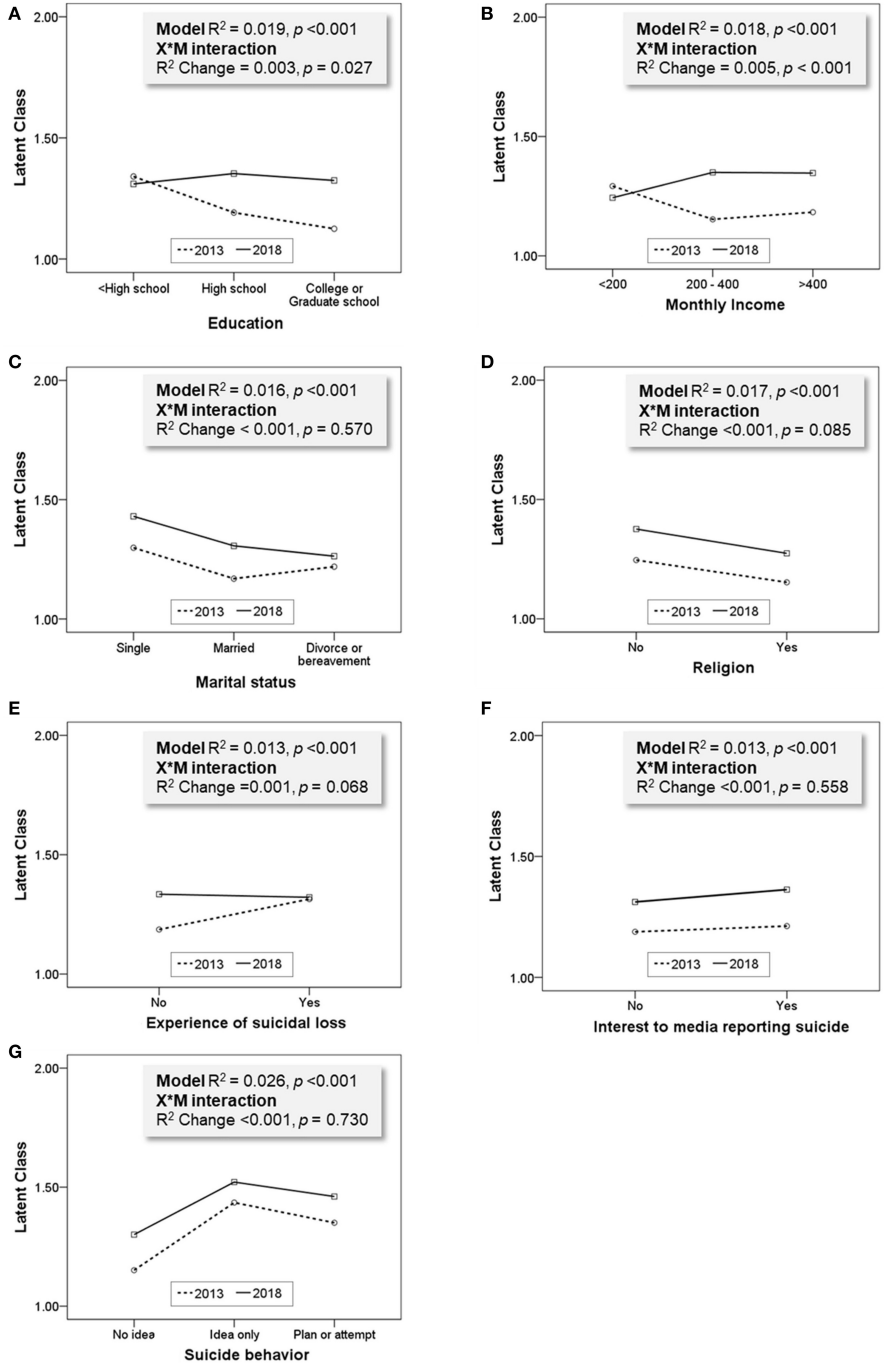
Moderating effect of each model. Age and sex were adjusted as covariates in each model. **(A)** Education (0 = < High school, 1 = High school, 2 = College or graduate school), **(B)** Monthly income (10^4^ KRW, 0 = < 200, 1 = 200–400, 2 = >400), **(C)** Marriage (0 = Single, 1 = Married, 2 = Divorce or bereavement), **(D)** Religion (0 = No, 1 = Yes), **(E)** Experience of suicidal loss (0 = No, 1 = Yes), **(F)** Interest in media reporting suicide (0 = No, 1 = Yes), **(G)** Suicide (0 = No idea, 1 = Idea only, 2 = Plan or attempt).

To identify the moderating effect of survey year between sociodemographic characteristics and attitudes toward suicide, moderation model analyses were performed for each factor as described in Figure 3. The results are presented in Figure 4. The moderation effect of survey year for the models of education (R^2^ change =0.003, *p* = 0.027) and monthly income (R^2^ change =0.005, *p* = 0.027) were statistically significant. When entering all other sociodemographic factors as covariates, the moderation effect was consistently significant with education (R^2^ change = 0.004, *p* = 0.003) and monthly income (R^2^ change = 0.005, *p* < 0.001). Other factors were not moderated by survey year.

## 4. Discussion

This study classified participants into three groups through a LPA with sub-factors of attitudes toward suicide: the incomprehensible group with high resistance to suicide, the permissive group with acceptance of suicide, and the intermediate mixed group. Previous studies have reported that higher acceptance of suicide is associated with higher risk of suicide, while higher resistance to suicide is associated with lower risk of suicide (16, 18). In the present study, the permissive group showed a higher association with suicide behavior than the incomprehensible group. Therefore, the permissive group could be regarded as a potentially high-risk group for suicide, while the incomprehensible group could be regarded as a low-risk group for suicide (5). The composition of the groups became more acceptive of suicide from 2013 to 2018. The proportion of the incomprehensible group decreased in 2018 compared to 2013, while the proportion of mixed and permissive groups increased. This increased vulnerability to suicide may reflect the increase in Korea's suicide rate in 2018, when compared to the suicide rate in 2013 (2). Previous studies have also reported increased acceptance of suicide (6) and increasing suicide rates in the U.S. (30). In addition, the mixed group showed higher risk of suicidal plans or attempts than the incomprehensible group. These results reflect that the mixed group, which occupied the highest proportion among the three groups (52.8%), could have a latent risk of suicide. Therefore, a strategy of targeting the mixed group to change attitudes toward suicide could help solve the problem of high suicide rates in Korea in future.

According to the 2013 survey data, participants with less than high school education were more likely to be in the mixed (OR = 2.20, *p* = 0.019) or permissive groups (OR = 2.40, *p* = 0.013) than the incomprehensible group. This result might be reflected by the previous findings demonstrating that the lower the level of education, the higher the risk of suicide (31, 32). However, there are differences in the influence of education level on attitudes toward suicide according to the survey country (18). For example, highly educated Russians condemn suicide, while highly educated Koreans, Americans, and Swedes perceive suicide as acceptable (6, 9, 18, 33). Some studies have reported that education could promote individualistic values and expressions of thought. As a result, people tend to have more receptive attitudes toward suicide (6, 9, 34). The effect of education level was not statistically significant in the 2018 survey, and the moderating effect of survey year between education and latent class was significant. Therefore, this decreased influence of education might have been reflected by the increased level of mean education in the Korean population (8). Therefore, it is necessary to carefully observe the change in the influence of educational factors on attitudes toward suicide in the future.

One previous study reported a positive association between income levels and permissive attitudes toward suicide (16). In the present study, the medium income group was less likely to be in the mixed group in both years. Especially in 2018, the low-income group was less likely to be in the mixed (OR = 0.42, *p* = 0.012) and permissive (OR = 0.34, *p* = 0.002) groups, and these results were confirmed by the moderation analysis results (Figure 4B). These results reflect that people with higher incomes perceived suicide as more acceptable in 2018. Since Korea's gross national income per capita has increased since 2000 (35), the influence of income level on permissive attitudes toward suicide should be considered important in the future.

People who have experienced suicidal loss in social relationships perceive suicide as acceptable and are more likely to display suicide behavior (17, 19). In the present study, experiencing suicide among acquaintances and permissive attitudes toward suicide were significantly related in the 2013 survey; however, permissive attitudes were not linked to these factors in the 2018 survey: In 2018, regardless of the presence of suicidal loss experience, there was a tendency to perceive suicide as acceptable compared to 2013. However, the moderating effect of survey year between suicidal loss and latent class was not significant (R^2^ change = 0.001, *p* = 0.068). This tendency may be due to the increased exposure to indirect experiences related to suicide through new media, especially in 2018 (15, 36).

Celebrity suicide reports positively affect receptiveness to suicide more than suicide reports regarding non-famous people (15). Interest in media reports on suicide issues can provoke suicidal motivations and cause the Werther effect (13, 15, 37, 38). Consequently, Korea's government enacted guidelines for suicide reporting in 2013, and there was no statistically significant difference in suicide deaths from the Werther effect between 2013 and 2017 (39). However, suicide deaths due to the Werther effect have increased again in Korea since 2018 (36), which is due to reduced compliance with suicide report recommendation standards for the media (26.6% in 2013 to 6.9% in 2015), increased reports of suicide-related news by new social media platforms, and sensational reports of self-harm (40–42). In the present study, while the frequency of those interested in media reports of suicide was lower in 2018 (41.5%) than in 2013 (46.2%), those with interest in such media reports were more likely to appear in the mixed (OR = 1.58, *p* = 0.029) and permissive groups (OR = 1.52, *p* = 0.048) in the 2018 survey. This may be reflected in the increased exposure to the new-media and news that indiscriminately report celebrity suicide (12, 14, 17, 43). Therefore, there should be more effort to establish more strict reporting regulations and be cautious when reporting through new media in the future.

People who had higher religiosity perceived suicide as a psychiatric problem, not an individual's own right, and an incomprehensible behavior (11). The results of this study demonstrated the association between religion and individual receptive attitudes toward suicide in both survey years. Finally, previous studies have reported a correlation between negative attitudes toward suicide and age (6, 44), sex (45), and marital status (6); however, no significant relevance was identified in this study.

## 5. Conclusion

This study classified latent classes according to attitudes toward suicide using a LPA and identified the meaningful sociodemographic characteristics of each group. Nevertheless, it has some limitations. First, data were analyzed separately for 2013 and 2018, and the effects of sociodemographic factors on attitudes toward suicide were compared by survey year. However, the participants surveyed in each year were independent groups, and changes in the influence of each factor are unlikely to reflect individual changes over time. In addition, the weight of the population was not adjusted in this study; thus, the results of our study are limited in representing the general Korean population. Second, as this was a cross-sectional study, causal inferences between sociodemographic factors and attitudes toward suicide could not be determined; therefore, further longitudinal studies are required in the future. Third, psychological factors that could affect attitudes or suicidal ideation, such as depressive symptoms, anxiety, or insomnia, were not included in this survey. Fourth, as the present study was conducted using a face-to-face interview method, participants may have underreported their symptoms and experiences. Fifth, in addition to suicide deaths, sudden deaths of close relations or member of family could affect an individual's depression level, attitude toward suicide, and suicide related behavior. However, we could not include this factor in the survey. Sixth, for determining the socioeconomic status, education and occupational groups might be more important than income level. Even though we included education level in this analysis, the occupational group analysis could not be conducted because of limited data.

Despite these limitations, these results have several strengths and implications. First, to our knowledge, the present study is the first to cluster groups according to attitudes toward suicide. The association between attitudes toward suicide and the intensity of suicidal ideas has been reported in previous studies (16–18). In particular, those who have permissive attitudes toward suicide were more likely to have suicidal ideas. Therefore, considering the latent subtypes of suicide attitudes classified in this study, we expected to be able to classify potential suicide risk level, which could be a useful indicator for the high-risk suicide-related behavior group. In addition, by identifying the significance of sociodemographic characteristics for each survey year in the classified group, this study analyzed changes in the influence of the changing demographic structure. Therefore, the results of this study have clinical implications and could aid in establishing suicide prevention policies to prepare for the changing demographic characteristics in the future.

## Data availability statement

The original contributions presented in the study are included in the article/supplementary material, further inquiries can be directed to the corresponding author.

## Ethics statement

The studies involving human participants were reviewed and approved by Institutional Review Board of Seoul National University Hospital (IRB No. 1810-062-979) and Kangwon National University Hospital (IRB No. KNUH-2013-06-007-001). Written informed consent for participation was not required for this study in accordance with the national legislation and the institutional requirements.

## Author contributions

HL: conceptualization, formal analysis, and writing—original draft. SJR, CHKP, JHY, and KS: conceptualization and writing—review and editing. MJK: data curation and writing—review and editing. J-IP: conceptualization and methodology. YMA: conceptualization, methodology, writing—review and editing, and supervision. All authors read and approved the final manuscript.
